# Hydrogen Sulphide modulating mitochondrial morphology to promote mitophagy in endothelial cells under high‐glucose and high‐palmitate

**DOI:** 10.1111/jcmm.13223

**Published:** 2017-06-13

**Authors:** Ning Liu, Jichao Wu, Linxue Zhang, Zhaopeng Gao, Yu Sun, Miao Yu, Yajun Zhao, Shiyun Dong, Fanghao Lu, Weihua Zhang

**Affiliations:** ^1^ Department of Pathophysiology Harbin Medical University Harbin China; ^2^ Bio‐Pharmaceutical Key Laboratory of Heilongjiang Province Harbin China

**Keywords:** diabetes, Parkin, hydrogen sulphide, mitophagy, mitochondrial fission/fusion

## Abstract

Endothelial cell dysfunction is one of the main reasons for type II diabetes vascular complications. Hydrogen sulphide (H_2_S) has antioxidative effect, but its regulation on mitochondrial dynamics and mitophagy in aortic endothelial cells under hyperglycaemia and hyperlipidaemia is unclear. Rat aortic endothelial cells (RAECs) were treated with 40 mM glucose and 200 μM palmitate to imitate endothelium under hyperglycaemia and hyperlipidaemia, and 100 μM NaHS was used as an exogenous H_2_S donor. Firstly, we demonstrated that high glucose and palmitate decreased H_2_S production and CSE expression in RAECs. Then, the antioxidative effect of H_2_S was proved in RAECs under high glucose and palmitate to reduce mitochondrial ROS level. We also showed that exogenous H_2_S inhibited mitochondrial apoptosis in RAECs under high glucose and palmitate. Using Mito Tracker and transmission electron microscopy assay, we revealed that exogenous H_2_S decreased mitochondrial fragments and significantly reduced the expression of p‐Drp‐1/Drp‐1 and Fis1 compared to high‐glucose and high‐palmitate group, whereas it increased mitophagy by transmission electron microscopy assay. We demonstrated that exogenous H_2_S facilitated Parkin recruited by PINK1 by immunoprecipitation and immunostaining assays and then ubiquitylated mitofusin 2 (Mfn2), which illuminated the mechanism of exogenous H_2_S on mitophagy. Parkin siRNA suppressed the expression of Mfn2, Nix and LC3B, which revealed that it eliminated mitophagy. In summary, exogenous H_2_S could protect RAECs against apoptosis under high glucose and palmitate by suppressing oxidative stress, decreasing mitochondrial fragments and promoting mitophagy. Based on these results, we proposed a new mechanism of H_2_S on protecting endothelium, which might provide a new strategy for type II diabetes vascular complication.

## Introduction

Diabetes mellitus is firmly established as a major threat to human health due to its severe complications in the cardiovascular system 1. Endothelial cell dysfunctions (ECD) play an important role in diabetic cardiovascular complications, which can result in the impairment of vasodilation, impairment of angiogenic properties and promotion of thrombus formation 2.

Accumulating evidence indicates that hyperglycaemia and hyperlipidaemia induced by type II diabetes could increase the production of ROS, increasing cell surface adhesion molecule expression and inflammatory changes that contribute to micro‐ and macrovascular damages 3. Meanwhile, mitochondria are the main source of ROS production. Mitochondria are morphologically dynamic organelles, undergoing constant fission and fusion events. There are many intracellular and extracellular signals that regulate mitochondrial fusion and fission, including oxidative stress, mitochondrial membrane potential collapse, mitochondrial DNA injury and apoptosis 4. Some studies demonstrated that the change in mitochondrial morphology was closely associated with the occurrence of mitophagy, which maintained mitochondrial homoeostasis 5, 6. Mitophagy is mediated by several proteins such as Parkin, PINK1, Mfn2 and Nix. The accumulation of PINK1 on the outer mitochondrial membrane (OMM) allows it to phosphorylate Parkin 7. Parkin, whose latent ubiquitin ligase activity becomes unmasked along the way owing to its phosphorylation by PINK1 8, 9 and its interaction with phospho‐ubiquitin 10, 11, is recruited to the mitochondria through association with the phosphorylated ubiquitin chain 12, 13, 14. Activated Parkin promotes the ubiquitination and subsequent degradation of many OMM proteins 15, 16. During this process, Parkin‐decorated mitochondria progressively cluster towards the perinuclear region to form mito‐aggresomes, which by virtue of their association with lysosomal components are removed by an autophagy‐dependent manner 17.

H_2_S is a newly found gas transmitter that has a strong antioxidative effect 18. In mammalian tissues, the biosynthesis of H_2_S is catalysed by the pyridoxal‐5‐phosphate‐dependent enzymes including cystathionine‐β‐synthetase (CBS) and cystathionine‐γ‐lyase (CSE). In the cardiovascular system, H_2_S is mainly catalysed by CSE. Studies have shown that H_2_S has the antioxidative effect. It has been reported that H_2_S is capable of suppressing ROS production and that ROS scavengers inhibit apoptosis. At 37°C and pH 7.4, more than 80% of H_2_S molecules dissolve in surface waters and dissociate into H^+^, HS^−^ and S^2−^ ions. HS^−^ is powerful one‐electron chemical reluctant and presents a remarkable capacity to scavenge ROS 19, 20. Therefore, we propose that exogenous H_2_S could protect RAECs from apoptosis through suppressing oxidative stress and promoting mitophagy under high glucose (HG) and palmitate condition.

## Materials and methods

### Cell culture and treatment

RAECs, purchased from Chinese Academy of Sciences Cell Bank, were grown as monolayers at a density of 5 × 10^4^ cells/cm^2^ in Dulbecco's modified Eagle's medium (DMEM) and incubated at 37°C in humidified air containing 5% CO_2_. The medium contained 10% calf serum, 100 units/ml penicillin and 100 μg/ml streptomycin. 2 days after seeding, the cultured RAECs were randomly divided into the following groups and treatments: control group (low glucose, LG, 5.5 mM), high glucose (HG, 40 mM) + palmitate (Pal, 200 μM), HG+ Pal+ NaHS (100 μM), HG+Pal+Mito‐TEMPO (2 μM, an inhibitor of mitochondrial reactive oxygen species), HG+Pal+Mdivi‐1 (50 μM, an inhibitor of Drp1), HG+Pal+bafilomycin A1 (100 nm, an inhibitor of autophagy) and HG+Pal+NaHS+ bafilomycin A1. Drugs were added directly in cultured medium for 48 hrs. RAECs treated with high glucose and palmitate classically mimic the endothelium in hyperglycaemia and hyperlipidaemia 21, 22.

### Detection of H_2_S in RAECs using H_2_S probe 7‐azido‐4‐methylcoumarin

The fluorescence intensity of H_2_S in RAECs was tested using 7‐azido‐4‐methylcoumarin (C‐7Az, Sigma‐Aldrich, USA), which has been proved to selectively respond to H_2_S. RAECs were incubated with 50 μM C‐7Az PBS for 30 min., followed by washing of the cells with PBS for three times. Visualization of the turn‐on fluorescence response of C‐7Az to H_2_S in RAECs was carried out using a fluorescence microscope (Olympus, XSZ‐D2, Japan). These results confirmed that excitation fluorescence imaging could be used to detect H_2_S through the triggered fluorescence response of C‐7Az.

### Western blotting analysis of total protein extraction from RAECs

RAECs were homogenized in 0.5 ml of RIPA buffer prior to being transferred into small tubes and rotated for 1 hr at 4°C. Solubilized proteins were collected after centrifugation at 12,000 *g* for 30 min. The protein concentration of each sample was quantified using the BCA Protein Assay kit. Polyacrylamide gels (12%) were used for protein testing, and equal amounts of proteins were applied and then electrotransferred onto a PVDF membrane (Millipore, USA). The non‐specific proteins on membranes were blocked with 5% non‐fat dried milk for 2 hrs at room temperature. Membranes were incubated with primary antibodies against anti‐CSE (1:1000, Proteintech, USA), anti‐SOD (1:1000, Proteintech, USA), anti‐β‐actin (1:1000, Proteintech, USA), anti‐Bcl‐2 (1:1000, Proteintech USA), anti‐Bax (1:1000, Proteintech, USA), anti‐cleaved‐caspase‐3 (1:1000, Proteintech, USA), anti‐cleaved‐caspase‐9 (1:1000, Proteintech, USA), anti‐Mfn2 (1:1000, Proteintech, USA), anti‐Fis1 (1:1000, Proteintech, USA), anti‐phospho‐Drp1/Drp1 (ser616, 1:1000, CST), anti‐Parkin (1:1000, CST), anti‐LC3B (1:1000, Proteintech, USA), anti‐Nix (1:1000, Proteintech, USA), anti‐beclin 1 (1:1000, Proteintech, USA), overnight at 4°C, respectively. Membranes were washed and then incubated with antimouse/anti‐rabbit IgG antibody at a 1:5000 dilution for 1 hr at room temperature. The specific complex was visualized using ECL plus Western blotting detection system. Densitometry was carried out with image processing and analysis by ImageJ, and the data were expressed as relative units.

### Mitochondrial ROS and cellular ROS level analysis

Mitochondrial ROS production was measured using MitoSOX Red mitochondrial superoxide indicator (Invitrogen, USA). RAECs were loaded with 5 μM MitoSOX Red at 37°C for 15 min. Red fluorescence was measured at 583 nm following excitation at 488 nm using a fluorescence microscope (Olympus, XSZ‐D2, Japan). Intracellular ROS contains oxidative radicals and superoxide anions. The oxidative radical levels were examined using the DCFH‐DA staining method based on the conversion of non‐fluorescent DCFH‐DA to the highly fluorescent DCF upon intracellular oxidation by ROS. RAECs were seeded on coverslips and incubated (45 min., 37°C, in the dark) in serum‐free media containing DCFH‐DA (10 μM) in the presence of control, high glucose and NaHS. After incubation, the conversion of DCFH‐DA to the fluorescent product DCF was measured using a spectrofluorometer with excitation at 484 nm and emission at 530 nm. Background fluorescence (conversion of DCFH‐DA in the absence of cells) was corrected by the inclusion of parallel blanks. The level of intracellular superoxide anions was detected by dihydroethidium (DHE). RAECs were incubated in serum‐free media containing DHE at 37°C for 30 min. DHE can be converted into ethidium by superoxide anions, and it showed red fluorescence at 535 nm. Cells were then incubated with Hoechst 33,342 for nuclear localization. The fluorescence intensity was analysed by ImageJ software in at least four fields of view and normalized by cell numbers.

### Cellular reactive oxygen species detection

RAECs were cultured in 96‐well black‐wall/clear‐bottomed microplates and plate cells overnight in growth medium at 10,000–40,000 cells/100 μl. Cellular reactive oxygen species were measured according to the assay protocol (Cellular Reactive Oxygen Species Detection Kit; Abcam, USA). 100 μl/well of ROS Red working solution was added to the cell plate and incubated in 5% CO_2_ at 37°C for 1 hr. Cells were treated with 20 μl of 11‐fold test compounds in PBS for 30 min., 2, 4 hrs. For control wells (untreated cells), equivalent amount of compound buffer was added. The fluorescence increase at Ex/Em = 520/605 nm was monitored with bottom read mode.

### Measurement of SOD activity and glutathione content

RAECs were treated with high glucose and palmitate (200 μM), NaHS and Mito‐TEMPO for 48 hrs, and then total protein was prepared. Superoxide dismutase (SOD) and glutathione (GSH) in the supernatant were measured using a spectrophotometer (Jiancheng Institute of Bioengineering, Nanjing, China). All assays were conducted according to the kit instructions.

### Hoechst 33342 / PI staining for apoptosis assay

Cells were seeded and treated for 48 hrs in 24‐well plates, washed three times with PBS, then incubated with 20 μg/ml Hoechst staining buffer for 15 min. at 37°C in the dark and then incubated with 20 μg/ml propidium iodide (PI) for 10 min. The percentage of apoptotic cells was observed by a fluorescence microscope (Olympus, XSZ‐D2, Japan).

### Analysis of mitochondrial transmembrane potential

Changes in mitochondrial transmembrane potential were assessed using the lipophilic cationic probe 5, 5′, 6, 6′‐tetrachloro‐1, 1′, 3, 3′‐tetraethyl‐imida‐carbocyanine iodide (JC‐1). RAECs were seeded and treated for 48 hrs at 37°C. After experimentation, cells were loaded with 2 μM JC‐1 (Invitrogen) at 37°C in the dark for 30 min. and washed three times with cold PBS. Green fluorescence reflected the monomeric form of JC‐1, and red fluorescence reflected the aggregated form. The cells were monitored using a fluorescence microscope (Olympus, XSZ‐D2, Japan).

### Detection of opening of mitochondrial permeability transition pore

The opening of mitochondrial permeability transition pore (mPTP) was measured by co‐culture of calcein‐AM with CoCl_2_. RAECs were treated for 48 hrs and incubated with calcein‐AM (2 μM) and CoCl_2_ (5 mM) at 37°C for 30 min. Calcein‐AM can enter into the mitochondria where it was converted into calcein. When mPTP was open, Co^2+^ entered into mitochondria. The fluorescence intensity of calcein was analysed by ImageJ.

### Mitochondrial isolation

Isolation of mitochondrial protein from RAECs was performed according to the manufacturer's protocol (Beyotime, Nantong, China). RAECs were washed twice with ice‐cold PBS, resuspended in lysis buffer (mmol/L: 20 Hepes/KOH, pH 7.5, 10 KCl, 1.5 MgCl_2_, 1.0 sodium EDTA, 1.0 sodium EGTA, 1.0 dithiothreitol, 0.1 PMSF and 250 sucrose) and then homogenized by an homogenizer in ice/water. After removing the nuclei and cell debris by centrifugation at 1000 *g* for 10 min. at 4°C, the supernatants were further centrifuged at 10,000 *g* for 10 min. at 4°C. The resulting mitochondrial pellets were resuspended in lysis buffer. The supernatants and mitochondrial fractions were stored at −80°C.

### Mitochondrial fragmentation

Using Mito Tracker staining (Beyotime) to observe the mitochondrial morphology, RAECs were seeded in 35‐mm culture dish and treated with different reagents and 200 nM Mito Tracker for 30 min. in a 37°C incubator containing 5% CO_2_ and then washed with PBS. A fluorescence microscope (Olympus, XSZ‐D2, Japan) was used for visualization and to determine the fluorescence intensity. We subtracted the background from the acquired images; the images were then filtered, and binary operations were applied to identify mitochondrial segments using ImageJ (NIH, Bethesda, MD, USA). Continuous mitochondrial structures were counted, and the number was normalized to the total mitochondrial area to obtain the mitochondrial fragmentation count (MFC) for each of 25 or more randomly selected cells, as described previously. Cells with greater fragmentation exhibit a higher MFC. The lengths of mitochondria were measured using NIS‐Elements software and scored as follows: fragmented (globular, <2 μm diameter); intermediate (2–4 μm long); and filamentous (>4 μm long). Approximately 200 cells were analysed, and the experiments were performed in triplicate by two individuals.

### Observation of mitochondrial ultrastructure by transmission electron microscopy

RAECs were washed with PBS and trypsin without EDTA and collected. The precipitates were obtained by centrifugation at 956 g for 10 min. and resuspended by PBS. RAECs were immersed immediately in fixative (3.0% glutaraldehyde buffered in 0.1 M sodium cacodylate, pH 7.2). Following 1–2 days of storage, specimens were raised in PBS, postfixed in cacodylate‐buffered 1% osmium tetroxide, dehydrated in ethanol series and embedded in Poly/Bed 812. Zeiss SUPRA55‐VP was used for observation of mitochondrial ultrastructure, including mitophagosomes and mitochondrial morphology.

### Mitochondrial autophagosome detection

RAECs were cultured in 24‐well plate. Mitochondrial autophagosomes were detected according to the assay protocol (mitophagy detection kit, Dojindo, Japan). Mtphagy Dye accumulates in intact mitochondria, is immobilized on it with chemical bond and exhibits a weak fluorescence from the influence of surrounding condition. When mitophagy is induced, the damaged mitochondria fuse to lysosome and then, Mtphagy Dye emits a high fluorescence. After incubation of cells with 100 nmol/l Mtphagy Dye working solution at 37°C for 30 min., cells were treated with HG+Pal, HG+Pal+NaHS, HG+Pal+Mito‐TEMPO, HG+Pal+bafilomycin A1 and HG+ Pal+NaHS+bafilomycin A1 for 48 hrs. Bafilomycin A1 was used as control to identify mitochondrial autophagosome. Then, cells were incubated at 37°C for 30 min. with 1 μmol/l Lyso Dye working solution to observe the co‐localization of Mtphagy Dye and lysosome. The mitophagy phenomenon and the fusion of mitochondria with lysosome were observed by on a fluorescence microscope (Olympus, XSZ‐D2, Japan).

### Immunoprecipitation assay

RAECs were collected as described above. The protein concentration of each sample was quantified using the BCA Protein Assay kit. Each sample was reacted with agarose (Protein G Plus Agarose, Santa) at 4°C for 20 min. and centrifuged at 10,000 *g* for 3 min. Agrose and antibody (Parkin, Ubiquitin) were added into the supernatants and incubated overnight. Immunoprecipitates were collected by centrifugation at 10,000 *g* for 5 min. and washed three times with lysis buffer, each time repeating centrifugation step above. Its purity was confirmed by Western blotting analysis with antibody PINK1, and Mfn2.

### Immunostaining

Cells were grown on culture dish, to label mitochondria, and 200 nM Mito Tracker^®^ Red CMXRos (Invitrogen) was added to the medium for 30 min. at 37°C, followed by a quick wash in PBS and fixation. Cells were fixed in 4% paraformaldehyde for 30 min. and then permeabilized with 0.5% Triton X‐100 for 30 min. Coverslips were blocked with 5% BSA for 1 hr at 37°C. Cells were incubated with anti‐Parkin antibody at 4°C overnight and washed three times with PBS, followed by incubation for 1 hr with anti‐rabbit IgG. Analysis and photomicrography were carried out with Zeiss LSM 510 inverted confocal microscope.

### siRNA transfection

RAECs (80% confluent) were treated according to the manufacturer's instructions with Parkin short interfering RNAs (siRNAs) (mouse; Santa Cruz Biotechnology, USA) for 72 hrs to inhibit Parkin expression. Transfection of RAECs by siRNA was achieved using Lipofectamine 2000 (Invitrogen). In brief, Parkin siRNA and control siRNA with the transfection reagent were incubated for 20 min. to form complexes, which then were added to plates containing cells and medium. The cells were incubated at 37°C in a CO_2_ incubator for further analysis.

### Statistical analysis

Data are expressed as the mean ± standard error (S.E.M.) Statistical analysis was analysed by one‐way anova. Differences between individual groups were analysed using Student's *t*‐test. *P* < 0.05 was considered statistically significant, and *P* < 0.01 was considered very significant.

## Results

### Exogenous H_2_S improved H_2_S level in RAECs

**Figure 1:**
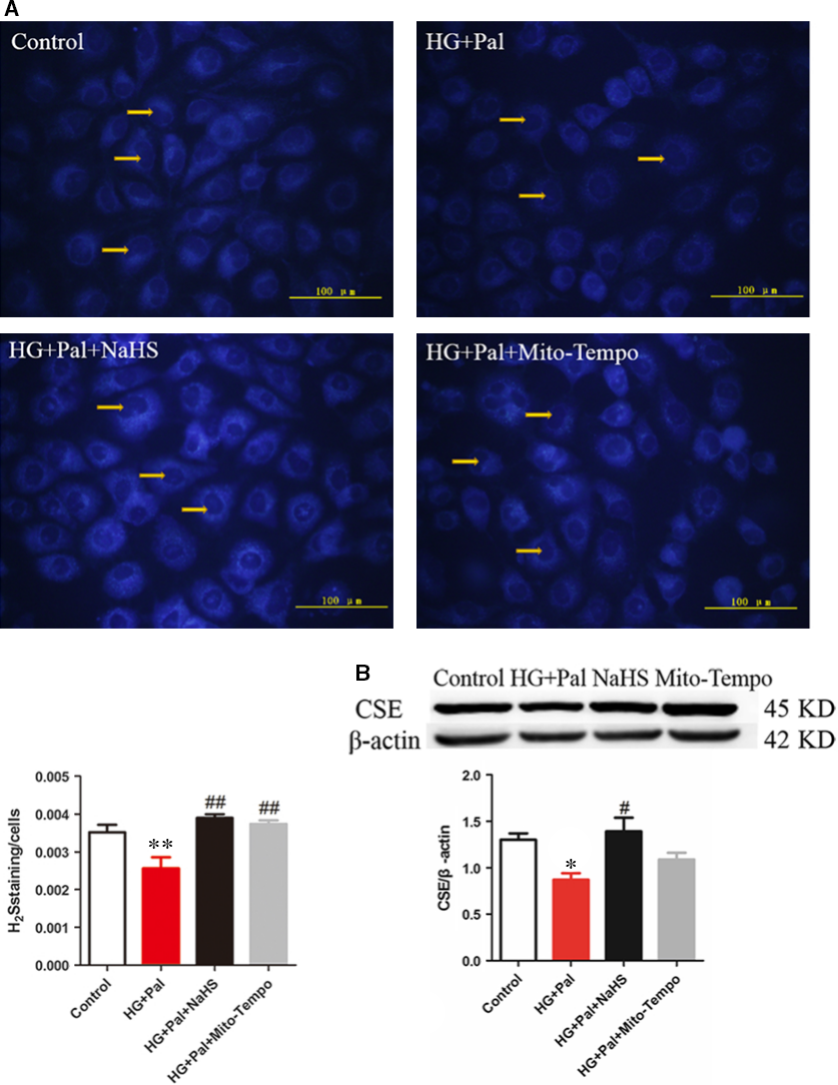
H_2_S content and the level of CSE were decreased under hyperglycaemia and hyperlipidaemia states. (**A**) RAECs were treated with 40 mM glucose, 200 μM palmitate, 100 μM NaHS and 2 μM Mito‐Tempo for 48 hrs, and the H_2_S level were detected. The fluorescence of H2S was observed by fluorescence microscope, and the nuclei were pointed out by the arrow. (*n* = 4, **P < 0.01 *versus* control group. ^##^ *P* < 0.01 *versus* HG+Pal group). (**B**) The CSE level of RAECs was tested by Western blotting. (*n* > 5, *P < 0.05 *versus* control group, ^#^ *P* < 0.05 *versus* HG+Pal group).

H_2_S is an important gaseous signal molecule in arteries, regulating the function of endothelial cells. RAECs with the treatment of HG and palmitate were used to imitate hyperglycaemia and hyperlipidaemia 21. Firstly, we used H_2_S probe 7‐azido‐4‐methylcoumarin to test the H_2_S content in RAECs. The H_2_S content was significantly decreased in the HG+Pal group, and its level was recovered by NaHS and Mito‐TEMPO treatment, an inhibitor of mitochondrial reactive oxygen species treatment (Fig. 1A). To further observe the H_2_S production in RAECs, the expression of CSE, the H_2_S production enzyme, was tested. We found that the CSE expression in HG+Pal group was significantly decreased, whereas it was increased by NaHS and Mito‐TEMPO treatment (Fig. 1B). The H_2_S production of RAECs was impaired by HG+Pal, which suggested that H_2_S might participate in endothelial cell injuries induced by hyperglycaemia and hyperlipidaemia.

### Exogenous H_2_S suppressed the production of ROS in RAECs

**Figure 2:**
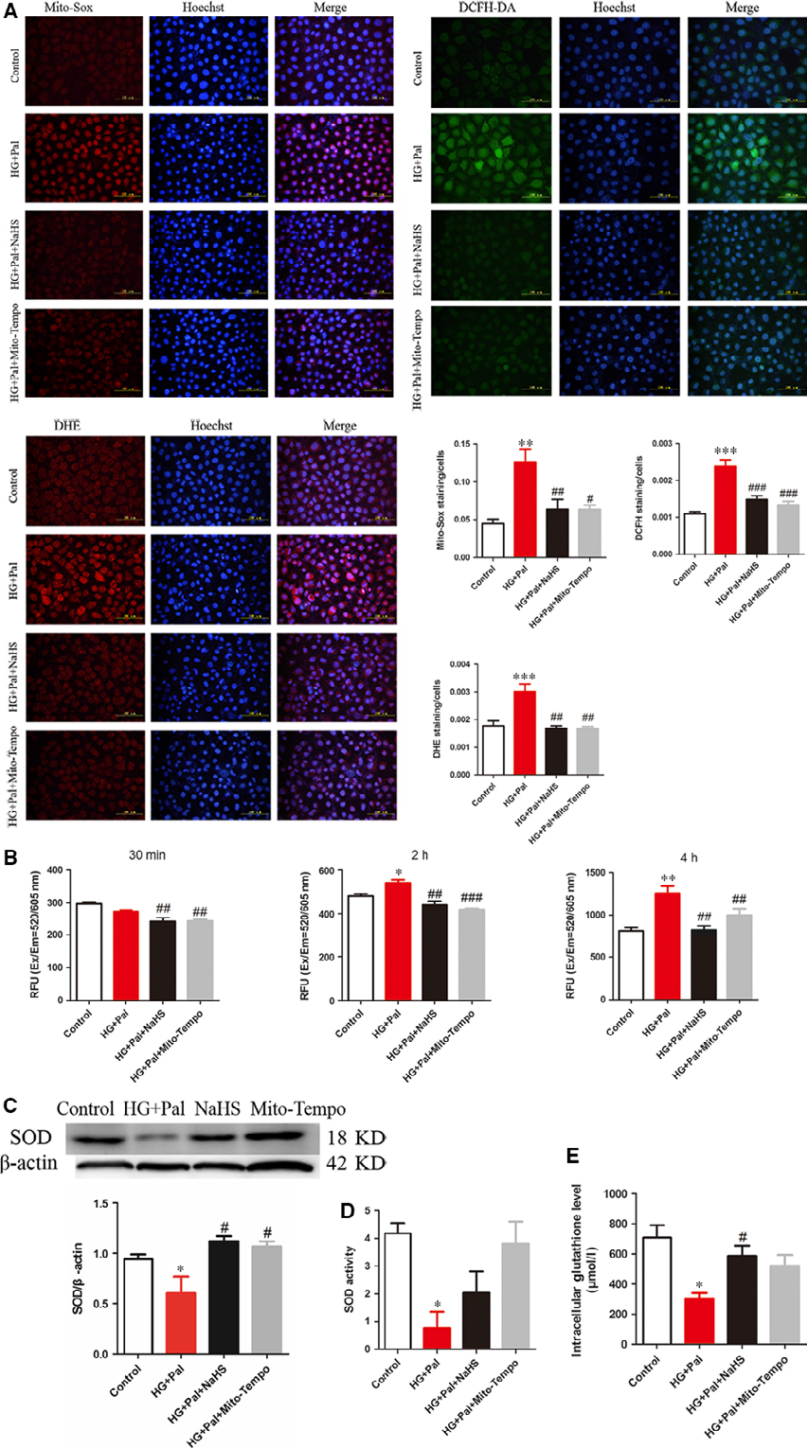
Exogenous H_2_S suppressed the production of ROS. (**A**) Mitochondrial and intracellular ROS were detected by MitoSox, DCFH, DHE, and the nuclei were labelled by Hoechst (blue fluorescence). (*n* = 4, **P < 0.01 *versus* control group, ***P < 0.001 *versus* control group, ^#^ *P* < 0.05 *versus* HG+Pal group, ^##^ *P* < 0.01 *versus* HG+Pal group, ^###^ *P* < 0.001 *versus* HG+Pal group). (**B**) ROS content was measured by ROS test kit in three different time‐points (30 min., 2 and 4 hrs). (*n* = 3, *P < 0.05 *versus* control group, **P < 0.01 *versus* control group, ^##^ *P* < 0.01 *versus* HG+Pal group, ^###^ *P* < 0.001 *versus* HG+Pal group). **(C, D, E**) The expression and activity of SOD and activity of GSH were measured. (*n* > 3, *P < 0.05 *versus* control group, ^#^ *P* < 0.05 *versus* HG+Pal group).

To investigate whether exogenous H_2_S could affect the ROS production in RAECs under HG+Pal condition, the MitoSox, DCFH and DHE were used to detect mitochondrial ROS, cellular superoxide anion and cytoplasmic H_2_O_2_, respectively. The results revealed that exogenous H_2_S suppressed the production of mitochondrial ROS and cellular superoxide anion and cytoplasmic H_2_O_2_ (Fig. 2A). To further confirm the antioxidant effect of exogenous H_2_S, we measured ROS content in three different time‐points (30 min., 2 and 4 hrs). We found that the ROS production increased with the time in HG+Pal group and exogenous H_2_S and Mito‐TEMPO significantly eliminated the ROS level (Fig. 2B). Furthermore, the activity and expression of SOD, an enzyme of ROS scavenger, were also improved by NaHS and Mito‐TEMPO (Fig. 2C and D). The intracellular antioxidant, glutathione, was increased in NaHS group (Fig. 2E). These results suggested that H_2_S is an efficient antioxidant for RAECs.

### Exogenous H_2_S inhibited mitochondrial apoptotic pathway of RAECs

**Figure 3:**
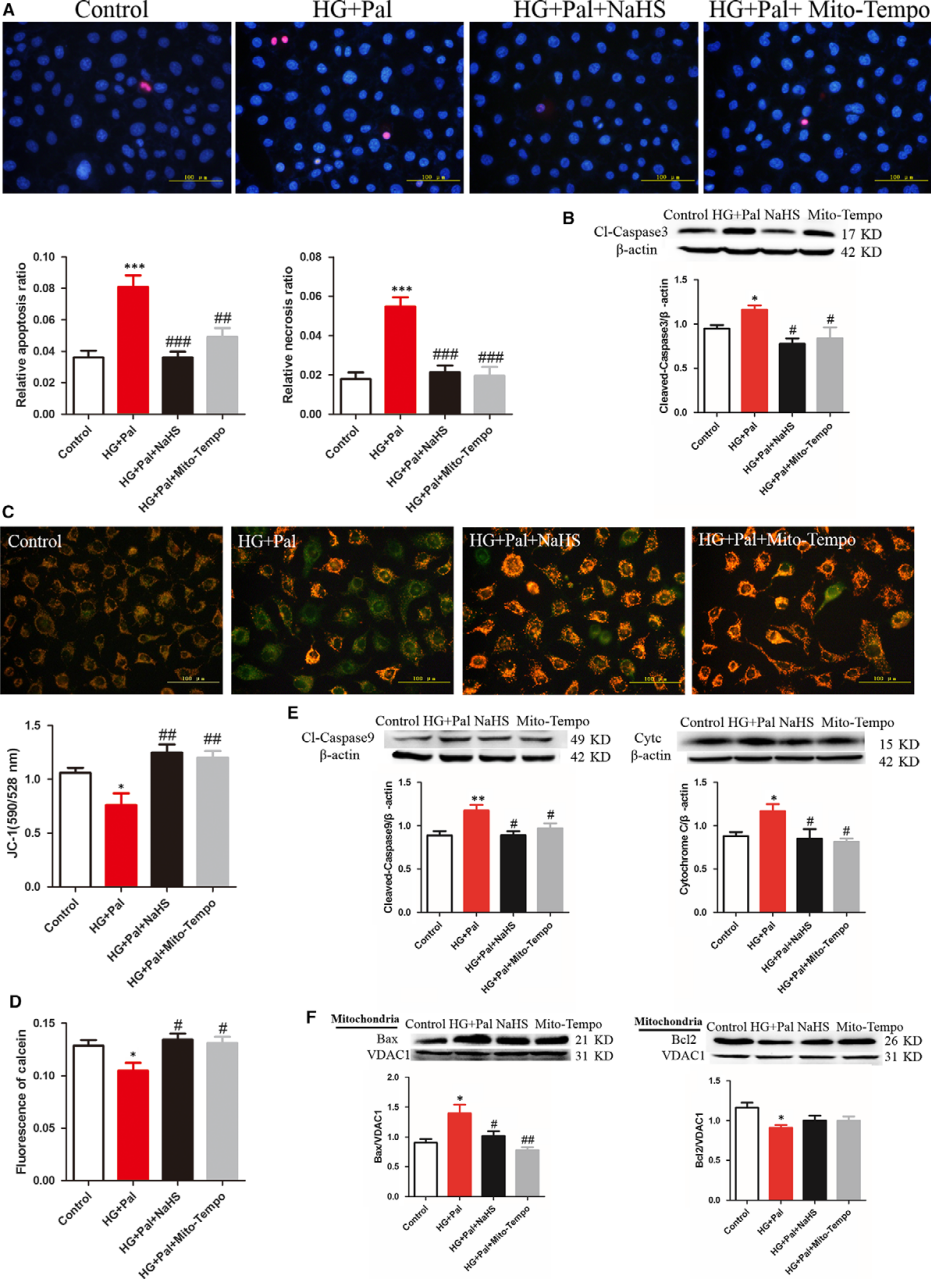
Exogenous H_2_S improved mitochondrial apoptosis of RAECs. (**A**) The apoptotic cells and necrotic cells were stained with Hoechst/PI assay. The quantity of positive cells was measured at least 200 cells in three different experiments. (*n* > 3, ***P < 0.001 *versus* control group, ^##^ *P* < 0.01 *versus* HG+Pal group, ^###^ *P* < 0.001 *versus* HG+Pal group). (**B**) The expression of Cl‐caspase3, the apoptotic marker, was tested by Western blotting. (*P < 0.05 *versus* control group, ^#^ *P* < 0.05 *versus* HG+Pal group). (**C**) JC‐1 assay was used to examine mitochondrial membrane potential. (*P < 0.05 *versus* control group, ^##^ *P* < 0.01 *versus* HG+Pal group). (**D**) The state of MPTP channel was examined by calcein assay. (*P < 0.05 *versus* control group, ^#^ *P* < 0.05 *versus* HG+Pal group). (**E**) Two markers of mitochondrial apoptosis, cleaved caspase‐9 (Cl‐caspase9) and cytochrome C (Cytc), were detected by Western blotting. (**P < 0.01 *versus* control group, ^#^ *P* < 0.05 *versus* HG+Pal group). (**F**) Bax and Bcl2, mitochondrial apoptotic markers, were also detected by Western blotting (*P < 0.05 *versus* control group, ^#^ *P* < 0.05 *versus* HG+Pal group, ^##^ *P* < 0.01 *versus* HG+Pal group).

Oxidative stress is a main reason for RAECs apoptosis. To investigate whether exogenous H_2_S could inhibit apoptosis, Hoechst/PI assay was used to examine the apoptotic cells. Our results showed that the apoptotic and necrotic cells in the HG+Pal group were significantly increased compared with the control group. NaHS treatment markedly attenuated the apoptotic rate (Fig. 3A). And the expression of cleaved caspase‐3 (Cl‐caspase3), the apoptotic marker, was also reduced by exogenous H_2_S, which supported the anti‐apoptotic effect of exogenous H_2_S (Fig. 3B). Mito‐TEMPO had the same effect as exogenous H_2_S on apoptosis, which suggested that mitochondria played a pivotal role in regulating the apoptosis of RAECs. Meanwhile, hyperglycaemia and hyperlipidaemia can also induce mitochondrial injury that is characterized by changes in mitochondrial membrane potential. JC‐1 assay was used to examine mitochondrial membrane potential. As shown in the results, the mitochondrial membrane potential was obviously collapsed in the HG+Pal group (green fluorescence and the red‐to‐green ratio were significantly decreased compared with the control group). Mitochondrial membrane potential was improved by NaHS and Mito‐TEMPO treatment (Fig. 3C). To further test why mitochondrial membrane potential was changed, the state of mPTP was examined by calcein assay. The result showed that exogenous H_2_S and Mito‐TEMPO suppressed the opening of mPTP induced by HG+Pal (Fig. 3D). The alteration in mitochondrial membrane potential could result in mitochondria inducing apoptosis. Thus, two markers of mitochondrial apoptosis, cleaved caspase‐9 (Cl‐caspase9) and cytochrome C (Cytc), were detected, and we found that exogenous H_2_S and Mito‐TEMPO decreased the expression of the two markers compared with HG+Pal group (Fig. 3E). Another couple of mitochondrial apoptotic markers, Bax and Bcl2, were also detected. The alterations in Bax and Bcl2 were ameliorated by NaHS and Mito‐TEMPO treatment (Fig. 3F). These results indicated that the protective effect of exogenous H_2_S on apoptosis of RAECs was attributed to its suppression of mitochondrial ROS production.

### Exogenous H_2_S protect mitochondria by modulating mitochondrial dynamics

**Figure 4:**
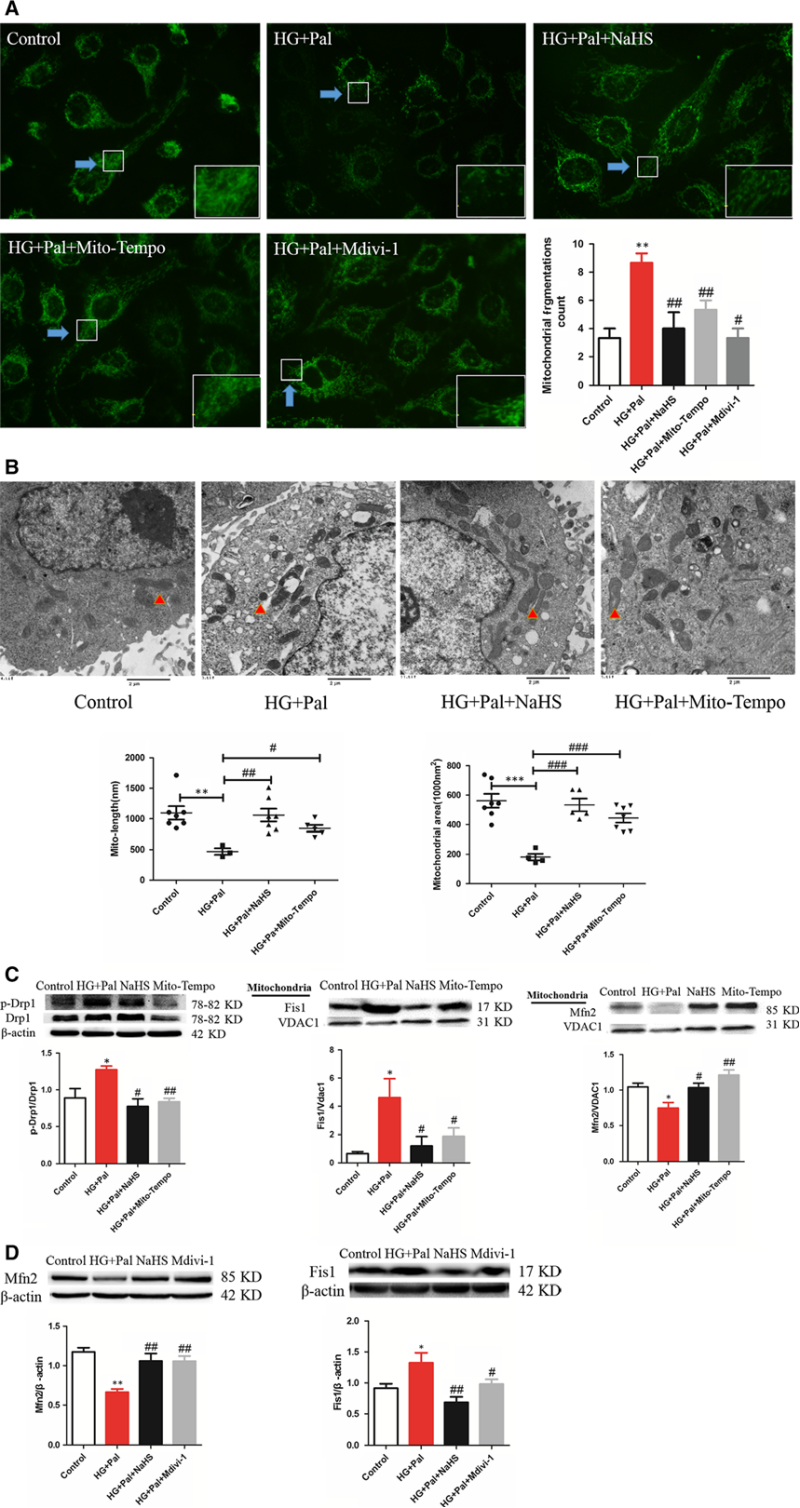
Exogenous H_2_S protected mitochondria through maintaining of mitochondrial dynamics. (**A**) The mitochondrial morphology of RAECs was measured by Mito Tracker green assay. (**P < 0.01 *versus* control group, ^#^ *P* < 0.05 *versus* HG+Pal group, ^##^ *P* < 0.01 *versus* HG+Pal group). (**B**) Transmission electron microscope was used to measure the size of mitochondria. (**P < 0.01 *versus* control group, ***P < 0.01 *versus* control group, ^#^ *P* < 0.05 *versus* HG+Pal group, ^##^ *P* < 0.01 *versus* HG+Pal group, ^###^ *P* < 0.001 *versus* HG+Pal group). (**C**) Three regulators associated with mitochondrial dynamics, p‐Drp1/Drp1, Fis1 and mitofusin (Mfn2), were detected by Western blotting. (*P < 0.05 *versus* control group, ^#^ *P* < 0.05 *versus* HG+Pal group, ^##^ *P* < 0.01 *versus* HG+Pal group). (**D**) The expression of Mfn2 and Fis1 was measured with the treatment of Mdivi‐1, an inhibitor of mitochondrial fission. (*P < 0.05 *versus* control group, ^#^ *P* < 0.05 *versus* HG+Pal group, ^##^ *P* < 0.01 *versus* HG+Pal group).

Mitochondria are highly dynamic organelles, undergoing constant fission and fusion events which are associated with its function and ROS production. Therefore, Mito Tracker assay was used to examine the mitochondrial morphology of RAECs. The results revealed that small mitochondria were accumulated in HG+Pal group compared with exogenous H_2_S and Mito‐TEMPO groups (Fig. 4A). To further investigate the mitochondrial morphology of RAECs, transmission electron microscopy was used to measure the size of mitochondria. We also found that small mitochondria were increased by HG+Pal treatment, which indicated that mitochondrial fission was broadly raised. This alteration was improved by NaHS and Mito‐TEMPO (Fig. 4B). To further investigate how exogenous H_2_S regulated mitochondrial dynamics, three regulating proteins associated with mitochondrial dynamics, p‐Drp1/ Drp1, Fis1 and mitofusin (Mfn2), were detected. Our results showed that high glucose and palmitate increased mitochondrial fission‐associated protein Fis1 and p‐Drp1/ Drp1 expression, whereas these proteins were down‐regulated with the treatment of exogenous H_2_S and Mito‐TEMPO. Mitochondrial fusion was detected by Mfn2 expression. High glucose and palmitate decreased Mfn2 expression that was increased by exogenous H_2_S and Mito‐TEMPO (Fig. 4C). Meanwhile, Mdivi‐1, an inhibitor of mitofission, had the same effect as exogenous H_2_S (Fig. 4D). Our results suggested that exogenous H_2_S promoted mitochondrial fusion and inhibited fission induced by HG+Pal.

### Exogenous H_2_S increased mitophagy in RAECs

**Figure 5:**
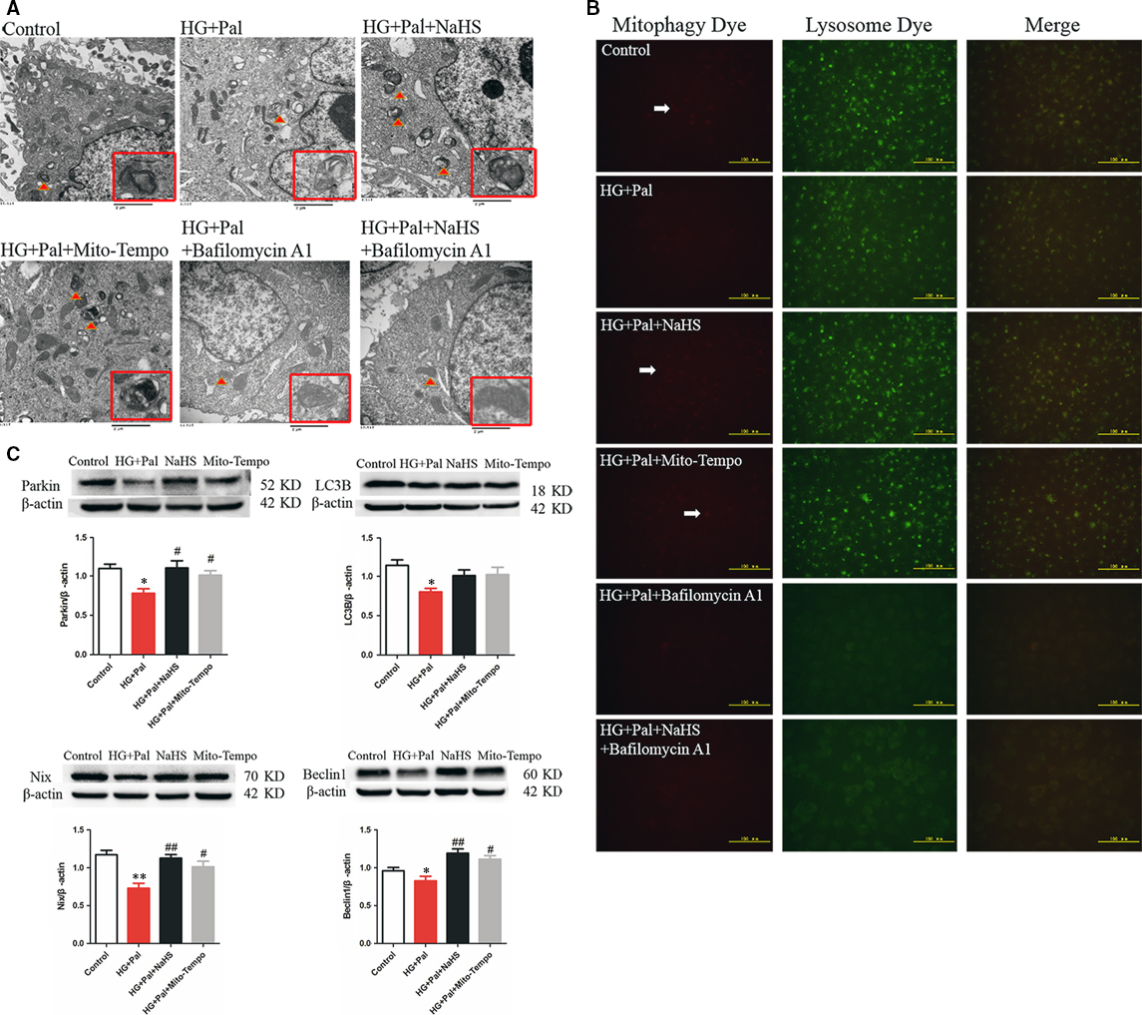
Exogenous H_2_S increased mitophagy. (**A**) Transmission electron microscope was used to detect the mitophagosomes. (**B**) Mitophagosomes were detected by mitophagy detection kit. Red fluorescence showed the mitophagosomes, and green fluorescence showed the fusion of mitophagosome and lysosome. (**C**) The expression of proteins associated with mitophagy, Parkin, LC3B, Nix and beclin 1, was also measured by Western blotting. (*P < 0.05 *versus* control group, ^#^ *P* < 0.05 *versus* HG+Pal group, ^##^ *P* < 0.01 *versus* HG+Pal group).

The mitochondrial ROS production is associated with the injured mitochondria which are mainly removed by mitophagy. Therefore, transmission electron microscopy and mitophagy detection kit were used to measure the mitophagy. We found that the number of mitophagosomes was highly increased by NaHS treatment (Fig. 5A, red arrows, Fig. 5B). The mitophagy was inhibited by the treatment of bafilomycin A1 through the observation of mitophagy by transmission electron microscopy (Fig. 5A). The result showed that exogenous H_2_S promoted the formation of mitophagosomes (red fluorescence) and the fusion of the damaged mitochondria with lysosome (green fluorescence) compared to HG+Pal and bafilomycin A1 groups (Fig. 5B). Proteins associated with mitophagy, Parkin, LC3B, Nix and beclin 1, were also detected. We found that the decrease in these four proteins induced with the treatment of high glucose and palmitate was improved by exogenous H_2_S and Mito‐TEMPO (Fig. 5C).

### Exogenous H_2_S up‐regulated mitophagy through activating PINK1/Parkin signalling pathway

**Figure 6:**
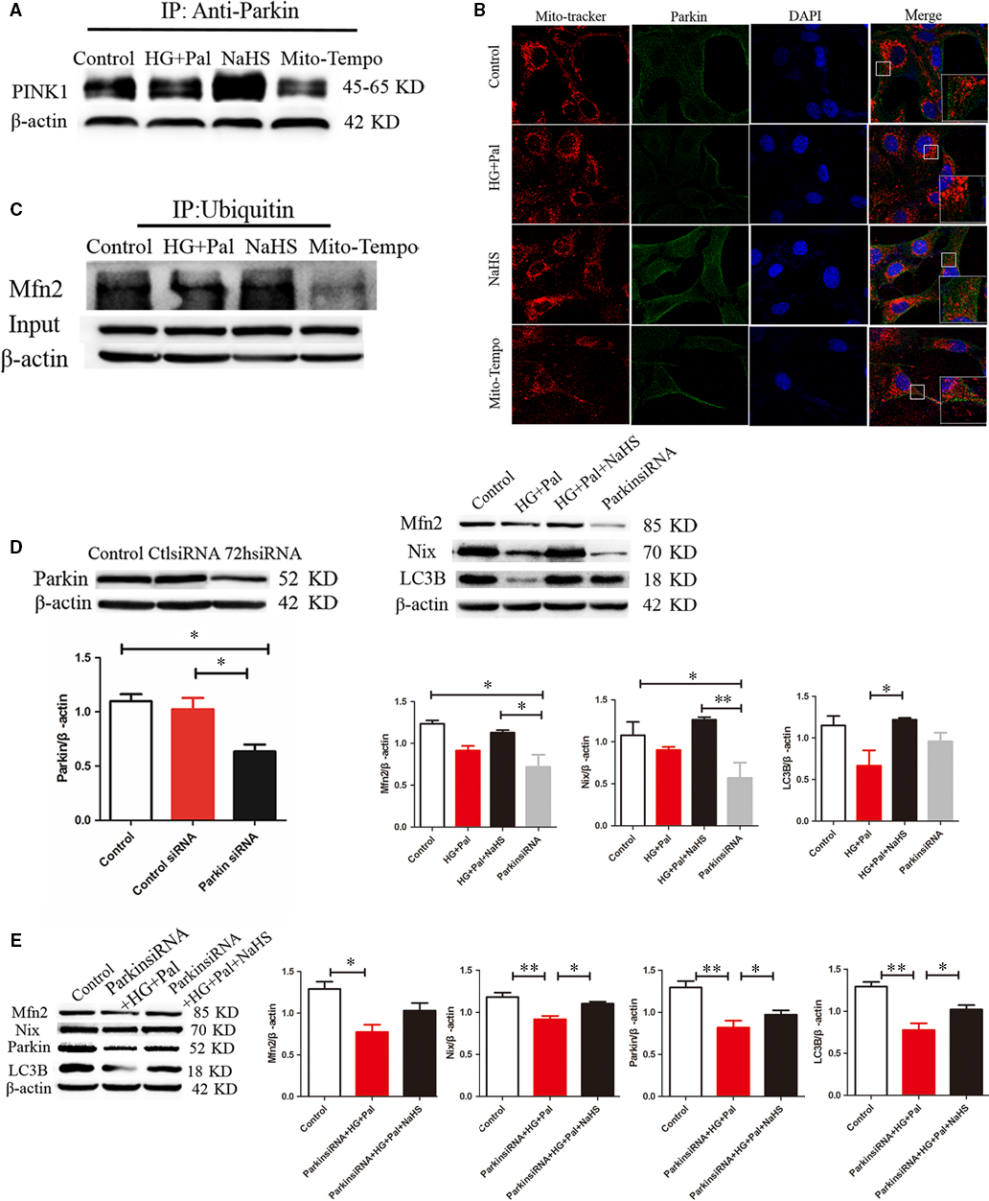
Exogenous H_2_S up‐regulated mitophagy through activation of PINK1/Parkin signalling pathway**.** (**A**) Immunoprecipitation assay was used to detect interaction between PINK1 and Parkin. (**B**) The expression of Parkin in mitochondria was detected by Mito Tracker (red fluorescence) and immunofluorescence for Parkin (green fluorescence) to investigate the recruitment of Parkin. (**C**) The level of ubiquitination of Mfn2 was detected by immunoprecipitation. (**D**) Parkin siRNA was used to knock down Parkin gene expression. The expression of Mfn2, Nix and LC3B was detected after Parkin knock‐down by Western blotting. (*P < 0.05, **P < 0.01). (**E**) Cells were treated by HG+Pal and HG+Pal+NaHS after knock‐down of Parkin by Parkin siRNA. The expression of Mfn2, Nix, Parkin and LC3B was detected by Western blotting. (*P < 0.05, **P < 0.01).

The interaction between PINK1 and Parkin plays a pivotal role in mitophagy. Thus, we used immunoprecipitation assay to detect the interaction between PINK1 and Parkin. We found that exogenous H_2_S enhanced the interaction between PINK1 and Parkin compared with high‐glucose and high‐palmitate groups (Fig. 6A). To further detect whether Parkin was recruited to the mitochondria, we used Mito Tracker (red fluorescence) and immunofluorescence for Parkin (green fluorescence) to investigate the recruitment of Parkin. The results showed that the expression and recruitment to the mitochondria of Parkin were increased by exogenous H_2_S (Fig. 6B). Mito‐TEMPO had little effects on recruitment of Parkin. Mfn2 is a key protein regulating mitophagy 23. The ubiquitination of Mfn2 by Parkin plays an important role in mitophagy. Therefore, we tested the ubiquitination of Mfn2 using immunoprecipitation. The result showed that exogenous H_2_S but not Mito‐TEMPO increased the ubiquitination of Mfn2 (Fig. 6C). To further investigate the effect of Parkin on mitophagy, Parkin siRNA was used to knock down Parkin gene expression. We found that the knock‐down of Parkin suppressed the expression of Mfn2, Nix and LC3B, which suggested that it eliminated mitophagy (Fig. 6D). After knock‐down of Parkin by Parkin siRNA transfection, exogenous H_2_S reversed the expression level of these proteins under hyperglycaemic and hyperlipidaemic states that further showed that exogenous H_2_S could improve mitophagy (Fig. 6E).

## Discussion

The results of current study reveal an effective protection of exogenous H_2_S on type II diabetes‐induced vascular endothelium injury and illuminate at least partly of its mechanism. Our results indicate that (*i*) exogenous H_2_S could attenuate the apoptosis of RAECs induced by high glucose and palmitate; (*ii*) exogenous H_2_S suppresses ROS production induced by high glucose and palmitate; (*iii*) exogenous H_2_S mediates mitochondrial dynamics and mitophagy in RAECs treated with high glucose and palmitate.

H_2_S has been proved to exert a wide range of physiological and cytoprotective functions in the biological systems. Our previous study has shown that exogenous H_2_S could attenuate the adhesivity of vascular endothelial cells in db/db^−^ mice 24. In our study, we used RAECs treated with high glucose and palmitate as hyperglycaemia and hyperlipidaemia cell models. We found that H_2_S production was decreased by high glucose and palmitate, which suggested the alteration in H_2_S content might be associated with vascular endothelium injury.

The role of H_2_S in oxidative stress has been one of the main focuses over years 25. Studies have also proved that H_2_S preserves the cellular GSH status and provides protection against oxidative damage in brain 26, 27, spinal cord 28 and heart 29, 30. We demonstrated that exogenous H_2_S could suppress both cytoplasmic and mitochondrial ROS production in RAECs treated with high glucose and palmitate. The specific mitochondrial ROS scavenger, Mito‐TEMPO, had the same effect as exogenous H_2_S on ROS production, which suggested that cytoplasmic ROS mainly root in mitochondrial ROS. Therefore, we deducted that the antioxidation of exogenous H_2_S might attribute to clearing the excessive mitochondrial ROS. Furthermore, previous studies have reported that diabetic vascular injury has mainly resulted from oxidative stress 31, and the excessive ROS production was interdepended on mitochondrial injury 32, 33. Meanwhile, mitochondrial injury reflects the change in mitochondrial membrane potential, and this change results from the abnormal opening of mPTP. We found that exogenous H_2_S could maintain the mitochondrial membrane potential through suppressing the abnormal opening of mPTP induced by high‐glucose and high‐palmitate treatment. Accordingly, we also demonstrated that exogenous H_2_S could suppress the accumulation of Bax on mitochondria through up‐regulating the expression of Bcl2, which indicated that exogenous H_2_S could inhibit mitochondrial apoptosis.

Mitochondrial morphology is maintained by mitochondrial dynamics which contains fission and fusion 34. There are many intracellular and extracellular signals that regulate fusion and fission events, including oxidative stress, membrane potential collapse, mtDNA injury and apoptosis 35. Mitochondrial fission often leads to the segregation of damaged components to one of the resulting mitochondria, producing one highly functional mitochondrion and another damaged mitochondrion with reduced membrane potential 36. However, oxidative stress can cause excessive mitochondrial fission, resulting in mitochondrial structural changes and dysfunction, and cell damages 37. We found that mitochondrial fragmentations or smaller mitochondria increased in high‐glucose and high‐palmitate group both through Mito Tracker and transmission electron microscopy assay, which indicated that mitochondrial fission was up‐regulated and this alteration was improved by exogenous H_2_S. Mito‐TEMPO had the same effects on mitochondrial morphology as exogenous H_2_S, which suggested that the role of exogenous H_2_S on mitochondrial dynamics might attribute to its antioxidative stress effect. The balance between mitochondrial fission and fusion is controlled by process‐specific proteins. Mitochondrial fusion is mediated by mitofusins (Mfn1 and Mfn2), which allows tethering of the opposing mitochondrial membranes. The fusion of two damaged mitochondria that harbour mutations in different genes can allow functional complementation to occur by the diffusion of RNA and protein components across the newly formed mitochondria, rescuing mitochondrial function 36. Accordingly, mitochondrial fission is regulated by proteins including Drp1 and Fis1 38. Drp1 is predominantly localized in cytoplasm. Only when recruited to the mitochondria does Drp1 associate with Fis1 localized to the outer mitochondrial membrane to form a complex that allows the mitochondrial fission. Our study demonstrated that exogenous H_2_S could up‐regulate the mitofusin, Mfn2, but down‐regulate the mitofission, p‐Drp1 and Fis1. So we concluded that exogenous H_2_S might have the capacity to inhibit mitofission but facilitate mitofusion.

Mitochondrial functions are also mediated by mitophagy, a special type of autophagy. The impaired mitochondria segregated by mitofission are cleared by mitophagy, which plays a crucial role in maintaining mitochondrial homoeostasis 6, 39, and they would like to be the main mitochondrial ROS source if cannot be cleared in time 40, 41. Our current study found that exogenous H_2_S could promote mitophagy in RAECs under high‐glucose and high‐palmitate condition, which suggested that exogenous H_2_S could facilitate the clearance of impaired mitochondria. The effect of exogenous H_2_S on suppressing ROS production might partly attribute to promoting mitophagy. Mitophagy is mediated parkin, a protein involved in all stages of the mitochondrial life cycle which is recruited to the mitochondria by PINK1 to trigger the mitophagy 11. The current study demonstrated that exogenous H_2_S but not Mito‐TEMPO increased the interaction between Parkin and PINK1, which indicated that exogenous H_2_S promoting mitophagy might not completely attribute to the antioxidative stress effect. Activated Parkin then promotes the ubiquitination of Mfn2, which is a role of the recruitment of Parkin to mitochondria 15, 16. We found that exogenous H_2_S but not Mito‐TEMPO increased the ubiquitination of Mfn2, which also indicated that exogenous H_2_S promoted mitophagy more efficiently than Mito‐TEMPO. The use of Parkin siRNA demonstrated that Parkin played a pivotal role in mitophagy, which suggested exogenous H_2_S promoted mitophagy efficiently. The effect of exogenous H_2_S on mitophagy implied that it might be an efficient way for H_2_S to suppress the mitochondrial ROS production and further oxidative stress and exogenous H_2_S might be more useful for the therapy of endothelial cell damage induced by type II diabetes.

## Conclusions

**Figure 7:**
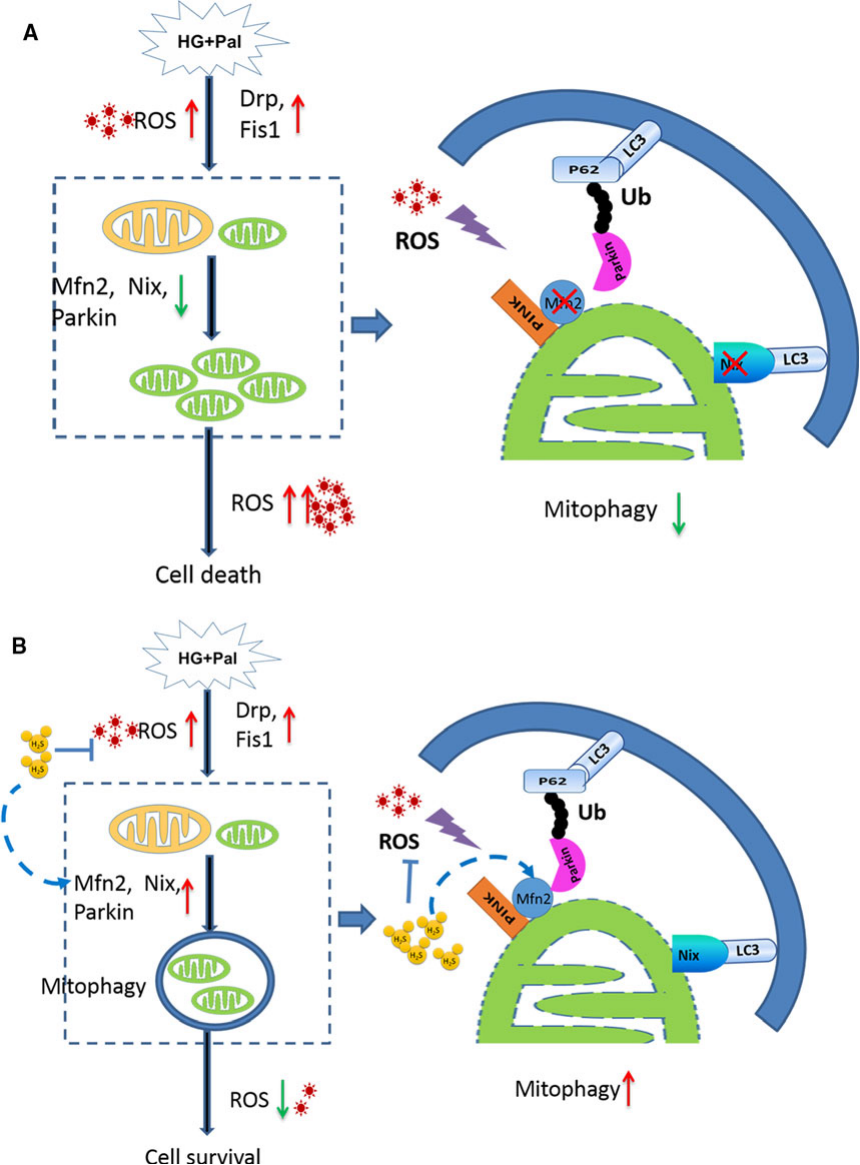
The protective effect of exogenous H_2_S on RAECs under HG and palmitate condition**.** (**A**) HG and palmitate increase ROS production, which up‐regulates mitochondrial fission but down‐regulates mitochondrial fusion. The expression of Mfn2 decreases, which reduces mitophagy. Thus, impaired mitochondria generated by mitochondrial fission cannot be cleared by mitophagy in time, which produces more ROS in turn. The ROS and damaged mitochondria finally induce mitochondrial apoptosis of RAECs. (**B**) Exogenous H_2_S is capable of suppressing ROS production and up‐regulating the expression of Mfn2 which recruits Parkin accumulating on the mitochondria and then triggers mitophagy. The damaged mitochondria are cleared by mitophagy, which reduces ROS production in turn and inhibits mitochondrial apoptosis of RAECs.

In summary, our current study demonstrated that exogenous H_2_S could protect RAECs against apoptosis under HG and palmitate by suppressing oxidative stress and modulating mitochondrial morphology by regulating mitophagy and mitochondrial dynamics (Fig. 7). Exogenous H_2_S not only rescues the endogenous of H_2_S but also protects arterial endothelial cells against cell death. Upon description of the mechanism conferred by exogenous H_2_S protection, it is possible to provide a new avenue for therapeutic opportunities for diabetes‐induced arterial endothelial injury.

## Conflict of interests

The authors declare that they have no conflict of interests.
